# Phenolic Content and Antioxidant Activity in *Trifolium* Germplasm from Different Environments

**DOI:** 10.3390/molecules24020298

**Published:** 2019-01-15

**Authors:** Aldo Tava, Łukasz Pecio, Roberto Lo Scalzo, Anna Stochmal, Luciano Pecetti

**Affiliations:** 1 CREA Research Centre for Animal Production and Aquaculture, viale Piacenza 29, 26900 Lodi, Italy; luciano.pecetti@crea.gov.it; 2 Department of Biochemistry and Crop Quality, Institute of Soil Science and Plant Cultivation, State Research Institute, ul. Czartoryskich 8, 24-100 Pulawy, Poland; lpecio@iung.pulawy.pl (Ł.P.); asf@iung.pulawy.pl (A.S.); 3 CREA Research Centre for Engineering and Agro-Food Processing, via G. Venezian 26, 20133 Milano, Italy; roberto.loscalzo@crea.gov.it

**Keywords:** antioxidant activity, flavonoids, isoflavones, phenolics, proanthocyanidins, *Trifolium*

## Abstract

Phenolics are important mediators in plant-environment interactions. The presence and concentration of phenolic compounds and their antioxidant activity were evaluated in leaves and flowers of a set of *Trifolium* species originating from contrasting environments encompassing lowland and mountain sites. The current germplasm proved a great reservoir of phenolic compounds, with different chemical structure and, possibly, diversified biological activity. Germplasm groups with specific phenolic composition were observed. In some cases, different patterns bore a taxonomic meaning. Lowland germplasm showed higher concentration of total phenolics in leaves than mountain accessions (50.30 vs. 34.19 mg/g dry matter (DM)), while the latter had higher concentration in flowers (114.16 vs. 57.44 mg/g DM). Outstanding concentration of isoflavones was observed in leaves of lowland germplasm (24.19 mg/g DM), and of both proanthocyanidins and flavonoids in flowers of mountain germplasm (53.81 and 56.62 mg/g DM, respectively). The pattern of phenolic composition in lowland and mountain germplasm was suggestive of different adaptive strategies. Three assays of antioxidant activity were tested, which were characterised by rather different reactivity towards phenolic composition. The scavenging activity was higher for leaf extracts of lowland germplasm, and for flower extracts of mountain germplasm. Besides identifying germplasm of interest, this study also suggested possible links between environmental factors and concentration and composition of phenolic compounds.

## 1. Introduction

The genus *Trifolium* includes a large array of species. Some of them represent very important forage crops worldwide, such as white clover (*T. repens* L.), and red clover (*T. pratense* L.) [1,2]. Other species have an agronomic relevance in specific areas, such as berseem clover (*T. alexandrinum* L.) and subterranean clover (*T. subterraneum* L.) in Mediterranean environments [3]. Wild forms of these and other cultivated species, as well as populations of many other *Trifolium* species occur in natural grasslands of diversified environments, where they can represent valuable feed resources [4,5,6].

Besides being rich in proteins, *Trifolium* species have been reported to contain a wealth of biologically active secondary metabolites [7], of which phenolic compounds are one of the main classes. Phenolic compounds have a wide range of structures, but they generally share a remarkable antioxidant activity [8]. Flavonoids are an important class of phenolics that includes compound groups such as flavones, flavonols and isoflavones, all characterized by a phenylbenzopyran chemical structure [8]. In recent decades, there has been an increasing interest on flavonoids in medical research owing to their useful properties, such as anti-inflammatory, estrogenic, antimicrobial, antiallergenic and antitumor activities [9]. The antioxidant activity, determined by their ability of decreasing free radical formation and scavenging free radicals and reactive oxygen species (ROS), is an asset of major interest for flavonoids [10,11]. 

The role of flavonoids and other phenolic compounds as protective dietary constituents (nutraceuticals) with their antioxidant capacity has also become an increasingly important area of research [12]. The potential benefit that a dietary intake of phenolics may produce in the prevention or reduction of degenerative diseases such as cardiovascular diseases and cancer has been reported [13,14]. Isoflavones are a group of flavonoids typical of some legume species only. They exhibit estrogenic activity and represent the main phytoestrogens of current interest as nutraceuticals and dietary supplements [15,16]. An antioxidant activity of possible physiological relevance was also reported for isoflavones such as genistein and daidzein [17].

Phenolic compounds, including flavonoids, have often been used as chemotaxonomic markers in plants [18]. These compounds were also examined in the genus *Trifolium*. Oleszek et al. [19] characterized the phenolic composition of over 50 *Trifolium* species, emphasizing similarities and differences among taxa for the concentration pattern of the main groups of phenolics. More recently, the isoflavone prunetin was proposed as a possible chemotaxonomic marker in snow clover (*T. pratense* L. subsp. *nivale* (Koch) Arcang.) [20].

Phenolics play a fundamental role in the interaction of plants with the environment, including their defense mechanisms against biotic and abiotic stresses and other adaptation processes [21,22]. It is well known that the presence of phenolics in plants and the subsequent antioxidant capacity can also be subjected to significant variation due to environmental conditions and to biotic and abiotic stresses [23,24]. Hence, the current study evaluated the presence and concentration of phenolic compounds, as well as their antioxidant activity in a set of *Trifolium* species originating from different Italian environments. The main objectives of this investigation were to identify germplasm of interest as potential source of phenolic metabolites, also in view of their possible exploitation for medical/nutraceutical purposes, and to assess any possible role of the environment on the phenolic composition of the species. 

## 2. Results

### 2.1. Germplasm Collection and Identification of Phenolics

**Figure 1:**
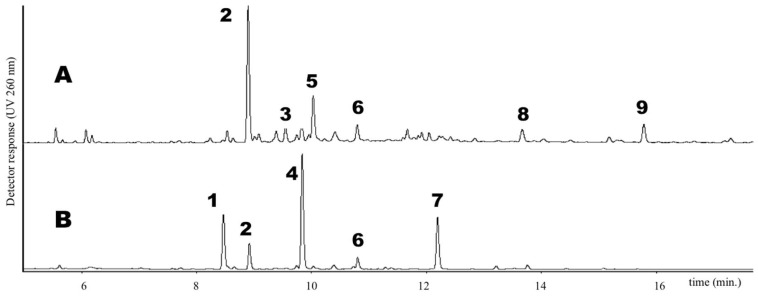
UPLC chromatogram of extracts from (**A**) leaves and (**B**) flowers of *T. repens* var. *sylvestre* (#13). **1**: myricetin-3-*O*-galactoside; **2**: quercetin-3-*O*-[xylosyl-(1→2)-galactoside]; **3**: kaempferol-3-*O*-[rhamnosyl-(1→6)- xylosyl-(1→2)-galactoside]; **4**: quercetin-3-*O*-galactoside; **5**: kaempferol-3-*O*-[xylosyl-(1→2)-galactoside]; **6**: kaempferol-3-*O*-galactoside; **7**: quercetin-3-*O*-galactoside-6′′-*O*-acetate; **8**: formononetin-7-*O*-glucoside; **9**: formononetin-7-*O*-glucoside-6′′-*O*-malonate.

**Figure 2:**
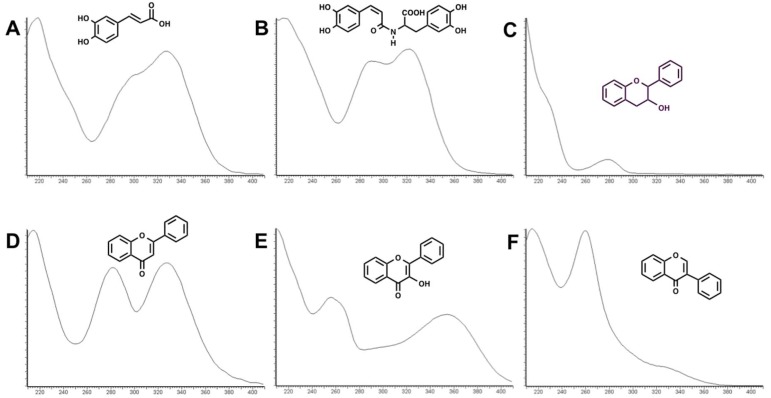
Characteristic UV spectra of the six groups of phenolics identified in *Trifolium* spp. **A**: phenolic acid; **B**: clovamide; **C**: flavanol; **D**: flavone, **E**: flavonol; **F**: isoflavone.

The list of *Trifolium* species used in this investigation is reported in Table 1. Both cultivated and natural populations from different environments (lowlands and mountains) were analyzed for their phenolic content. Phenolics occurring in the extract of the 14 *Trifolium* samples under investigation were separated by UPLC method (see Materials and Methods) and UV spectra were obtained using a photo diode array detector. As an example, the UPLC chromatogram of leaf and flower extracts of *T. repens* var. *sylvestre* is shown in Figure 1. Six groups of phenolics were identified based on their characteristic absorption spectra (Figure 2), namely: phenolic acids (Figure 2A), clovamides (Figure 2B), flavanols (Figure 2C), flavones (Figure 2D), flavonols (Figure 2E) and isoflavones (Figure 2F).

A comparison was made of their retention times and mass spectral data obtained in positive and negative mode with those of standard compounds or with compounds previously reported in literature for *Trifolium* spp. [19,20,25,26,27,28]. A tentative identification of phenolics was performed based on key fragment ions and other MS observations. For flavonoids and their glycosyl derivatives the loss of 162 *m/z* was indicative of hexose (glucose or galactose), the loss of 146 *m/z* was indicative of rhamnose, the loss of 132 *m/z* was indicative of pentose (xylose or arabinose). Moreover, the loss of 44 *m/z* in the negative ion mode and the loss of 86 *m/z* were indicative of the presence of a malonate. 

Branched *C*-glycosides were also investigated by the presence of characteristic ions [M − H − 60]^−^, [M − H − 90]^−^, and [M − H − 120]^−^ [25,26,27,28,29]. 

Along with clovamide (*N*-caffeoyl-l-DOPA), phenolic acids were detected in almost all *Trifolium* extracts in low amount and were mostly constituted by glycosyl derivatives of caffeic acid, ferulic acid and coumaric acid. 

Flavanols were identified in very low amount (< 0.3 mg g^−1^ dry matter) in some extracts, and identified as catechin/epicatechin and a catechin dimer. Flavones, flavonols and isoflavones represented the main bulk of flavonoid constituents of the extracts. The most abundant, tentatively identified, flavonoids in the 14 *Trifolium* samples are reported in Table 2 where their percentage amount in the whole extracts is indicated. All the identified compounds were quantitatively evaluated by appropriate standards (see Experimental).

Glycosyl and glycosyl malonate derivative of luteolin, together with lower amount of derivatives of apigenin and of the isoflavone biochanin A, were the most abundant compounds identified in *T. alexandrinum* (#1) extracts. In flowers, large amounts of quercetin galactoside and quercetin glucoside were also detected. All these compounds were previously reported in the species [30,31,32]. High amounts of glycosyl and glycosyl malonate derivative of flavones (quercetin in leaves and quercetin and kaempferol in flowers) and isoflavones (biochanin A and formononetin in leaves and flowers) were detected in red clover (#2 and #7), as previously reported in this widely investigated *Trifolium* species [19,20,33,34,35,36]. In snow clover (#3 and #12) extracts, glycosyl and glycosyl malonate derivative of flavones (quercetin) and isoflavones (formononetin and prunetin) were detected in large amounts in both leaves and flowers. The abundant presence of prunetin was reported to be a characteristic feature of this clover species [20]. Leaf extracts of *T. repens* (#4, #8 and #13, see Figure 1) featured high content of di- and trisaccharide derivative of flavonols quercetin and kaempferol, together with other minor compounds, as already reported [37,38,39,40]. Flowers were characterized instead by high amount of quercetin galactoside and its acetyl derivative, together with lower content of myricetin galactoside. Subterranean clover (#5 and #6) leaves and flowers confirmed the large presence of isoflavones biochanin A, genistein and formononetin glycosides and glycosyl malonate derivatives [28]. *T. alpinum* (#**9**) extracts showed a complex mixture of flavonoids. In leaves, they were mainly constituted by mono and diglycosides of quercetin, while kaempferol glycosides were detected in flowers. The presence of glycosides of a quercetin isomer (MW=302) was also detected. The very low amount of isoflavones assessed in this *Trifolium* species was inconsistent with previous results [26]. Di- and tri-saccharide derivatives of quercetin were observed in *T. badium* (#10) extracts. An unidentified flavonoid (MW = 462) was abundant in leaves, while a monoglycoside of luteolin/luteolin isomer (MW = 286) was detected in large amount in flowers. 

Leaves of *T. ochroleucum* (#11) mainly comprised mono- and di-saccharides of quercetin and, to a lesser extent, kaempferol. Quercetin and kaempferol glycosyl malonates were detected as the main constituents of flower extracts. Higher flavonoid content had been previously reported in *T. ochroleucum* leaves [19]. Quercetin and kaempferol diglycosides were abundant in *T. thalii* (#14) leaf extract, while the flower extract largely contained myricetin and quercetin glycosides. The isoflavone formononetin glycoside and its malonyl derivative were also detected in leaves.

### 2.2. Evaluation of Phenolic Compounds in the Trifolium Species in Relation to Their Antioxidant Activities

To facilitate the assessment of the biological activity of the different *Trifolium* extracts and to evaluate their antioxidant properties in relation to the phytocomplex (whole extract), the detected compounds were grouped into four distinct classes based on their chemical structure and biological properties, namely, phenolic acids, clovamides, isoflavones, and other flavonoids, this latter including flavanols, flavonols, and flavones. The presence of a fifth group of phenolic compounds, namely proanthocyanidins (or condensed tannins) was assessed by the butanol/HCl method [41], and evaluated with pelargonidin as a standard. Although with some limitations, this method was reported to allow for the most effective detection of proanthocyanidins [42,43,44]. Pelargonidin was selected as a standard because of its presence in all samples among anthocyanidins obtained from the acid-catalysed cleavage of the condensed tannins. Preliminary investigation performed by a cellulose bidimensional thin layer chromatography (2D TLC) of the obtained anthocyanidin (data not reported), showed the presence of pelargonidin, cyanidin and delfinidin. In all the analyzed samples, variation was observed for these compounds, with pelargonidin being one of the most detected compounds. 

Results of the quantitative evaluation of different phenolics in leaves and flowers of the 14 samples of clover under investigation are reported in Table 3, Table 4 and Table 5. 

Flower tissues featured higher concentration of total phenolics compared to leaves across all accessions (Table 3). However, differences between plant organs for individual phenolic group concentrations were not consistent, with no difference for phenolic acids and clovamides, higher concentration in leaves for isoflavones, and higher concentration in flowers for other flavonoids and for proanthocyanidins. The antioxidant activity was higher in flower than in leaf extracts according to each of the three applied assays.

It is widely accepted that leaves have higher amount of phenolic compounds than flowers, owing to the abundance of pathway precursors in leaves due to photosynthesis [45,46]. However, the current result of higher concentration of total phenolics in flowers than in leaves had several precedents in different folk-medicine species, such as yarrow, *Achillea millefolium* L. [47], pomegranate, *Punica granatum* L. [48], or white-weed, *Ageratum conyzoides* L. [49]. The facts that pigments are composed of flavonoids in most flowers, and that proanthocyanidins are produced by closely related branches of the flavonoid pathway using the same metabolic intermediates, make of no surprise the finding that flowers in our germplasm collection were particularly rich in these groups of phenolics. Abeynayake et al. [50] reported that white clover plants accumulate higher level of proanthocyanidins in flowers than in vegetative tissues. In addition to a possible ultraviolet (UV)-screening effect exerted by phenolics [22], a role for flavonoids was reported in the development of functional pollen [51], which may also explain the abundance of these compounds in flower tissues. Total phenolic concentration in flowers, especially as proanthocyanidins, fully supported higher antioxidant activity of flower compared to leaf extracts, no matter the applied scavenging assay. Nonetheless, greater sensitivity of the peroxyl radical scavenging for proanthocyanidins was evident, confirming the results in Jayaprakasha et al. [52], thus accounting for the current outstanding difference between leaf and flower extracts for this assay.

The 14 *Trifolium* samples differed significantly (*P* < 0.05) for leaf phenolic concentrations and antioxidant activity according to ANOVA (Table 4). The red clover cultivar Aiace (#2) was rich in phenolic acids, followed by the snow clover population #3 and the berseem clover experimental cultivar #1. Clovamides were found in highest concentration in red clover samples (#2 and #7) and they were also present in the snow clover populations #3 and #12 and the berseem cultivar #1. 

The subterranean clover populations #5 and #6 showed the highest concentration of isoflavones, followed by the two red clover samples (#2 and #7) and the two snow clover populations (#3 and #12). Three mountain species, namely, sulphur clover (#11), brown clover (#10) and alpine clover (#9) featured the highest concentration of other flavonoids, together with the berseem clover (#1). The two white clover accessions from lowlands (#4 and #8) and the two subterranean clover populations (#5 and #6) were characterized by the lowest concentration of other flavonoids. Proanthocyanidins were abundant in leaves of brown clover (#10) and berseem clover (#1) only. The leaf concentration of total phenolics exceeded the level of 60 mg g^−1^ dry matter in the two red clover accessions (#2 and #7), the subterranean clover population #5 and the berseem clover #1, while barely reaching the level of 10 mg g^−1^ dry matter in the two lowland white clover samples (#8 and #4).

Just as for the leaf samples, there was significant variation among accessions (*P* < 0.05) according to ANOVA for the flower phenolic concentrations and antioxidant activity (Table 5). However, accession patterns of phenolic concentrations were largely different in flowers compared to leaves. Only clovamides showed an outstanding consistency between flowers and leaves of exclusive presence in red clover (#2 and #7), snow clover (#3 and #12) and berseem clover (#1) germplasm (Table 4 and Table 5). Concentration of phenolic acids was highest in flowers of the red clover samples (#2 and #7), the snow clover population #3, the sulphur clover population #11 and the berseem clover cultivar #1. Isoflavone concentration (much lower in flowers than in leaves, see Table 3) was highest in the red clover accessions (#2 and #7), the snow clover population #3 and the subterranean clover population #6. The subterranean clover population #5, featuring the highest isoflavone concentration in leaves, was missing in the flower analysis, owing to too few flowers to be used in the chemical determinations. The brown clover population #10 showed the highest concentration of other flavonoids, followed by the Thal clover population #14, while the subterranean clover population #6 had remarkably low concentration of these compounds. Somewhat similar was the pattern of proanthocyanidin concentration, with highest values in mountain accessions (brown clover #10, Thal clover #14, snow clover #12, and white clover #13) and lowest one in the subterranean clover #6. The very high concentration of proanthocyanidins in the mentioned mountain accessions clearly contributed to their highest concentrations of flower total phenolics, with values exceeding 100 mg/g dry matter, while the subterranean clover population #6 was bottom ranking for flower total phenolics with a concentration of about 10 mg/g dry matter (Table 5).

Oleszek et al. [19] partitioned their *Trifolium* collection into groups according to the patterns of leaf phenolic composition. The present germplasm also featured accession groups with specific phenolic composition. In some cases, these patterns bore a taxonomic meaning. *T. subterraneum* and *T. pratense* (the latter, both as red clover and as snow clover subspecies) were characterized by high leaf concentration of isoflavones. Clovamides were restricted to red clover and, to a lesser extent, snow clover and berseem clover. Most alpine species and berseem clover were rich in flavonoids other than isoflavones. White clover (across the three evaluated taxonomic forms) featured the lowest leaf phenolic concentration.

The richness of leaf isoflavones in subterranean clover confirms previous results on this species [28]. Red clover isoflavone extracts are commercially available as nutraceuticals and they have been proposed as an alternative to hormone-replacement therapy [53]. Subterranean clover may represent an interesting new source of isoflavones, with higher concentration of these compounds and more diverse pattern of isoflavone composition compared to red clover [28].

As emphasized [19], attention should be paid for exploitation to those species comprising good concentration of different phenolic groups, as the presence of these groups may provide synergistic health effects of plant extracts.

Accession rankings for the antioxidant activity of leaf extracts according to the three assays were quite inconsistent (Table 4). The highest activity was observed in the two red clover samples (#2 and #7) for the Fremy’s salt scavenging, in the subterranean clover population #5, the red clover population #7 and the snow clover population #12 for the peroxyl radical scavenging, and in the brown clover population #10 and the red clover cultivar #2 for the superoxide anion scavenging. The inconsistency of accession ranking for the three assays was confirmed by the lack of significant pairwise correlation between assays based on leaf accession values (data not reported).

Unlike for the data on leaf extracts, there was some consistency of accession ranking between assays for the antioxidant activity of flower extracts (Table 5). In particular, the Thal clover population #14 and the subterranean clover population #6 were always top- and bottom-ranking, respectively, regardless of the scavenging assay. The mountain white clover population #13 also featured rather high antioxidant activity of flower extract with all three assays. Overall, there were moderately high positive pairwise correlations between scavenging assays, ranging from *r* = 0.74, *P* < 0.01 (Fremy’s salt vs. peroxyl radical) to *r* = 0.87, *P* < 0.01 (Fremy’s salt vs. superoxide anion).

The presence of diversified patterns of phytochemical composition, and the contemporary presence of structurally different groups of phenolics, such as isoflavones, other flavonoids and proanthocyanidins, that were likely to determine different responses to the antioxidant assays [54], justified the choice of three methods of antioxidant measurements. These assays were characterised, indeed, by rather different reactivity towards distinct free radical molecular probes, as successively confirmed by the results of the regression analysis described below. 

Lowland accessions had higher concentration of phenolic acids, clovamides and isoflavones, and lower concentration of other flavonoids and proanthocyanidins, than mountain accessions consistently in leaves and flowers. However, lowland germplasm showed higher concentration of total phenolics in leaves, while mountain accessions had higher concentration of total phenolics in flowers (Table 6). This inconsistency was mostly due to outstanding concentration of isoflavones in leaves of lowland germplasm, and of both proanthocyanidins and flavonoids other than isoflavones in flowers of mountain germplasm. An inconsistency of rankings between germplasm provenances was also largely observed for the antioxidant activity. Lowland accessions had, on average, higher scavenging activity of leaf extracts in two assays out of three, whereas the scavenging activity of flower samples was higher in mountain than in lowland germplasm regardless of the assay (Table 6).

As already anticipated, the high concentration of flavonoids (other than isoflavones) and proanthocyanidins, associated with higher antioxidant capacities, in flower tissues of mountain germplasm can be a hint of the photoprotection effect recognized to flavonoids and other phenolics [55], possibly exerted on the sensitive reproductive system [23]. It is indeed well known that UV levels increase with altitude, and mountain species must be provided with adequate protection. Accumulation of flavonoids in response to UV-B exposition was reported in silver birch, *Betula pendula* Roth [56], while UV-stress-adapted germplasm displayed particularly high constitutive or elicited levels of flavonoids and other phenolics in *Arabidopsis thaliana* (L.) Heynh. [57] and in white clover [38]. UV absorption is one of the UV-protective properties ascribed to flavonoids, which also include energy dissipation and antioxidant activities [55]. A role of free-radical scavenging in response to UV exposure can be postulated [58].

The pattern of phenolic composition in lowland germplasm was suggestive of different adaptive strategies. The abundance of isoflavones in lowland leaves, in particular, may indicate natural selection to decreased palatability for herbivores, or increased defence systems against invertebrate pests [59]. 

Table 7 summarizes the best linear models predicted for leaf and flower samples from the multiple regression analysis of the antioxidant activity on mean concentrations of groups of phenolic compounds within the *Trifolium* germplasm. The concentration of ‘other flavonoids’ and that of ‘proanthocyanidins’ consistently proved affected by multicollinearity in flowers, and for that reason only the former was retained in the regressions on flower data. Regardless of the scavenging assay and the plant organ, the adjusted *R^2^* of the models was only moderate at most, with the exception of the good prediction of the Fremy’s salt scavenging in leaves (*R^2^* = 0.934, *P* < 0.001). In both leaves and flowers, the peroxyl radical scavenging had a complex best fitting model, including most of the phenolic groups as regressors, whereas the Fremy’s salt and the superoxide anion scavenging showed simpler models for both plant organ extracts.

The concentration of clovamides had a consistent, positive regression coefficient in the three best models predicted for the antioxidant activity in leaves. Similarly, the concentration of the group of flavonoids other than isoflavones contributed positively to predicting the antioxidant activity with all assays in flowers (Table 7). 

The multicollinearity between the concentrations of flavonoids and proanthocyanidins prevented their simultaneous assessment as regressors in the prediction of the antioxidant activity. However, when taken individually in a simple correlation analysis with the three scavenging assays, the mean flower concentration of proanthocyanidins also proved positively correlated with the antioxidant activity, with correlation ranging between *r* = 0.63 (*P* < 0.05) with the peroxyl radical and *r* = 0.73 (*P* < 0.01) with the superoxide anion scavenging.

Although claimed as interesting antioxidant compounds, clovamides proved to be important but not critical for the antioxidant activity in cocoa, *Theobroma cacao* L. [60]. Subsequent findings indicated them as potent bioactive compounds with anti-inflammatory activity in human cells [61]. In the current study, clovamides appeared to have a conditional, positive role in determining the antioxidant activity of leaf extracts in the genus *Trifolium*. Our results are in line with those by Kolodziejczyk et al. [62], who reported that clovamide-rich extract from *T. pallidum* reduced the damage induced by oxidative stress to blood platelets and plasma.

A key role in determining the antioxidant activity in flowers of *Trifolium* germplasm was exerted instead by the classes of flavonoids (isoflavones excluded) and proanthocyanidins, which was fully justified by the strong antioxidant properties reported for these compounds [11,52,63]. As already mentioned, flower pigmentation and UV screening contribute to the abundance of flavonoids and proanthocyanidins in flower tissues.

This investigation confirmed the genus *Trifolium* as a great reservoir of phenolic compounds, with different chemical structure and, possibly, largely diversified biological activity. Such a wealth of potentially important metabolites is available for clinical and nutraceutical utilization. It is worth reminding that, unlike other species claimed for extraction of biologically active compounds, most *Trifolium* species have a well-established agronomic technique for their cultivation. This study also suggested possible links between environmental factors (stresses, in particular) and concentration and composition of phenolic compounds.

## 3. Materials and Methods 

### 3.1. Plant Material

Swards of red clover, white clover, subterranean clover, berseem clover and snow clover were sampled in the lowland site of Lodi [45°19′ N, 9°30′ E, 81 m above sea level (asl)], in the Po Valley plain, northern Italy (Table 1). Some of the materials belonging to these taxa were grown in plots at the experimental station of the Research Centre for Animal Production and Aquaculture (CREA-ZA). In particular, the red clover cultivar Aiace, the white clover cultivar Regal (a non-Ladino large-leaved type of *T. repens*) and one snow clover natural population from the Rhaetian Alps were sampled from one-year-old sown swards. Aiace and Regal are cultivars selected in Italy and the USA, respectively, and both are adapted to temperate, favorable climatic conditions, such as those experienced under the sub-continental climate of Lodi (847 mm long-term average annual rainfall, 12.2 °C average annual mean daily temperature, −1.3 °C average minimum daily temperature of the coldest month). Seed of the snow clover population from the Rhaetian Alps was collected at 1850 m asl and it was sown in the lowland site as part of a trial carried out in altitude-contrasting environments. Germplasm of the annual species berseem clover (the experimental cultivar Sintetica-E) and subterranean clover (two natural populations whose seed was originally collected in Sardinia) were sown at CREA-ZA for this study. As both species are specifically adapted to Mediterranean conditions, they were sown in Lodi at the end of the winter preceding the sampling season to avoid possible frost damages. Besides the plot-grown materials, two more populations were sampled from a century-old grassland in Lodi, belonging to locally-adapted ecotypes of red clover and Ladino white clover (*T. repens* L. var. *giganteum* Lagr.-Foss.), respectively. Other natural populations used in this investigation were identified and sampled in the Alpine region (Table 1). They included one small-leaved population of white clover (*T. repens* var. *sylvestre*), which encompassed, therefore, three different types in the study, and one population each of snow clover, alpine clover (*T. alpinum* L.), brown clover (*T. badium* Schreb.), sulphur clover (*T. ochroleucum* Huds.) and Thal clover (*T. thalii* Vill.). Identification of different taxa was made according to reference guides on the Alpine flora [64]. All the mountain populations except the sulphur clover were sampled in the Graian Alps above 1900 m asl. The sulphur clover population was sampled in the Cottian Alps at 1250 m asl. 

All the materials were sampled at the same phenological stage of full bloom. This stage occurred in spring (May) in the lowland site of Lodi, and in summer (July) in the mountain locations. Two samples of leaves and flowers were collected, respectively, from each sward, refrigerated and immediately brought to the laboratory. Leaf and flower samples were separately freeze-dried, finely powdered, defatted with chloroform and then used for the subsequent extractions. 

### 3.2. Extraction and Purification of Total Phenolics

Samples (100 mg) were extracted with 80% MeOH (10 mL) at 50 °C using an automated accelerated solvent extractor ASE 200 (Dionex, Sunnyvale, CA, USA) at a working pressure of 1500 psi. Extracts were evaporated to dryness under reduced pressure at 40 °C, re-dissolved in 5 mL of Milli-Q water (Millipore Corp., Billerica, MA, USA) and purified using a C18 Sep-Pak (360 mg, 55–105 μm) cartridge (Waters Associates, Milford, MA, USA) preconditioned with water. The cartridge was washed first with water (5 mL) to remove sugars and other water-soluble compounds, then with 80% MeOH (5 mL) to elute phenolics. Extracts were evaporated to dryness under reduced pressure at 40 °C, re-dissolved in 2 mL of 80% MeOH and then used for analysis. Three independent extraction and purification procedures were performed on each replicated sample, and the extracts were properly diluted before analysis. A portion of the extracts was also freeze-dried for subsequent antioxidant activity assays. 

### 3.3. Determination and Quantitation of Phenolic Composition

A Waters ACQUITY UPLC system equipped with a binary pump system, sample manager, column manager, photo diode array (PDA) detector (Waters Corp.) and coupled to a Waters ACQUITY TQD (tandem quadrupole mass detector) with an electrospray ionization (ESI) source was used. All data were acquired and processed using Waters MassLynx 4.1 and QuanLynx software. Chromatographic runs were carried out with a Waters BEH C18 column (100 mm × 1.0 mm i.d., 1.7 μm particles, 13 nm pore size) under a linear gradient of solvent A (H_2_O/0.1% HCOOH) and solvent B (CH_3_CN/0.1% HCOOH) as follows: 0.0–0.5 min (7% B), 8.0 min (25% B), 11.5 min (60% B), 12.0 min (80% B), 20 min (80% B). The flow rate was 0.19 mL/min, and the column temperature was 50 °C. A sample of 0.5 μL was injected for analysis. For MS detection, positive and negative ESI were used as ionization modes. Nitrogen was used as the desolvation and cone gas with flow rates of 800 L/h and 80 L/h, respectively. Argon was used as the collision gas at a flow rate of 2 mL/min. The MS parameters were as follows: Capillary 2.8 kV; extractor and radiofrequency voltage fixed at 3.0 V and 0.1 V, respectively; source and desolvation temperatures of 140 °C and 350 °C, respectively [20,28]. 

Phenolic compounds were identified by comparison of their UV absorption spectra, retention times and mass spectral data (positive and negative mode) with those of standard compounds, as well as with previously identified phenolic constituents of *Trifolium* spp. reported in the literature [19,20,25,26,27,28]. Individual compounds were quantified against reference standards at 260 nm. Astragalin, hyperoside, cymaroside, isoquercitrin, genistin, ononin, sissotrin, kaempferol, quercetin, apigenin, luteolin, myricetin, genistein, biochanin A, formononetin catechin, epicatechin were from Sigma-Aldrich (Milano, Italy). Concentration of glycoside malonates and acetates were calculated using the standard curves of the corresponding glycosides. Concentration of isoflavonoids without standards were calculated using the standard curves of ononin, while the concentration of other flavonoids was calculated using the calibration curves of isoquercitrin. Chlorogenic acid from Sigma-Aldrich was used to quantify clovamides and phenolic acids. For calibration curves, all standards were injected in triplicate in the range from 4.5 to 300 ng injection. 

Proanthocyanidins (condensed tannins) were assessed by the butanol/HCl method [41], and evaluated with pelargonidin hydrochloride from Sigma-Aldrich as a standard.

All samples were extracted in triplicate and results expressed in mg g^−1^ dry matter as mean of three independent analyses ± standard deviation. As an indication of the relative abundance of the main tentatively identified compounds, the percentage values of their content in the whole extracts were reported in Table 2. 

### 3.4. Antioxidant Activity

The purified freeze-dried extracts were subsequently treated at room temperature with 2.5 mL MeOH, 2.5 mL water, and acidified with 0.05 mL of 1N HCl. The mixture was vortexed for 60 s and centrifuged (5000× *g*, 5 min) and the supernatant directly used for antioxidant assays. The antioxidant activity was assessed by three different methods, aimed at understanding the potential action of the different groups of antioxidant compounds isolated and thoroughly characterized in *Trifolium* extracts. The three methods, described as follows, were the Fremy’s salt scavenging and the superoxide anion scavenging, both carried out by Electron Paramagnetic Resonance (EPR), and the scavenging of peroxyl radicals, performed with a spectrophotometric approach.

#### 3.4.1. Fremy’s Salt Scavenging

This EPR assay was performed using Fremy’s salt, potassium nitrosodisulfonate [(KSO_3_)_2_NO], a persistent water-soluble free radical that was successfully used in previous experiments investigating the antioxidant potential of fruit juices [65]. The reaction mixture contained 380 µL of 0.1 M acetate buffer, pH 4.5, and 100 µL of plant extract with 20 µL of 5.96 mM Fremy’s salt solution dissolved in acetate buffer, final concentration 0.24 mM. Blank reaction was composed as before, by replacing the plant extract by the pure extracting solution. The mixture was stirred and transferred into a 100-µL glass capillary tube, and the EPR spectra were recorded after 3 min at 25 °C using a MS 200 EPR spectrometer (Miniscope, Berlin, Germany) operating on the X-band. The instrument settings were the following: field modulation 100 KHz, modulation amplitude 1500 mG, field constant 60 s, center field 3350 G, sweep width 99.70 G, X-band frequency 9.64 GHz, MW attenuation 7 dB, and gain 5. Under these conditions, the typical triplet Fremy’s salt spectrum (1:1:1) was observed. The intensity of the EPR signal was measured at the height of the first line, with the resonance at 3336.6 G. The system was calibrated with solutions of gallic acid at known concentrations, hence the results were given as µmol of gallic acid equivalents (GAE)/100 g dried extract.

#### 3.4.2. Superoxide Anion Scavenging

The antioxidant potential towards O_2_^−•^ was based on the spin trapping of the radical generated by potassium superoxide (KO_2_) in dimethyl sulfoxide (DMSO) with the addition of 18-crown-6 ether to complex K^+^, using the previously performed and validated method by Picchi et al. [66], with some modifications. The spin trap reagent was 5,5-dimethylpyrroline-N-oxide (DMPO). Under these conditions, a typical DMPO-OOH adduct (1:1:1:1) was observed. The scavenging reaction mixture was 18-crown-6 ether/KO_2_ (1:1, 20 mM) dissolved in DMSO. A blank solution contained 100 µL of a 100 mM DMPO phosphate buffer solution 0.1 M, pH 7.4, 130 µL of superoxide solution and 10 µL of extracting solution. The solution with the scavenging compound was composed exactly as the blank, by replacing 10 µL of extracting solution with the equivalent volume of the *Trifolium* extract. The reaction time was 60 sec at 25 °C. EPR recording conditions were as follows: field set, 3350 G; scan range, 70 G; scan time, 120 s; modulation amplitude, 1200 mG; microwave attenuation, 5 dB; receiver gain, 8 × 100. The intensity of the EPR signal was measured at the height of the first spectrum line, with the resonance at 3326.1 G. The system was calibrated with solutions of gallic acid at known concentrations, hence the results were given as mmol of gallic acid equivalents (GAE)/100 g dried extract.

#### 3.4.3. Peroxyl Radical by Enzymatic Degradation of Linoleic Acid

In this method, the enzymatic peroxidation of linoleic acid, generating peroxyl radicals, was obtained by the addition of lipoxygenase (EC 1.13.11.12) with some modifications of the method described by Grossman and Zakut [67], successively checked and validated by Lo Scalzo et al. [68]. The peroxidation of linoleic acid was analyzed in the absence (blank) and presence (sample) of the assayed extracts by recording the increase in absorbance at 234 nm over 2 min at 25 °C after a 30 sec delay. The substrate was prepared by dissolving 40 µL of linoleic acid in 2 mL of absolute ethanol under nitrogen, followed by the addition of 10 µL of Tween 20. The solution was slowly mixed, then 40 mL of 0.05 M K_2_HPO_4_ were added, and the pH was adjusted to 9.0 with 0.05 M NaOH, the final substrate concentration resulting 3.21 mM. The lipoxygenase solution was freshly prepared by dissolving 11 mg of a lipoxygenase standard soybean extract (Sigma, St. Louis, MO, USA) in 16 mL of 0.1 M phosphate buffer (pH 7.0). In the sample test, the reaction solution was composed of 2.0 mL of 0.1 M phosphate buffer (pH 7.0), 0.2 mL of substrate (0.27 mM final concentration), 0.15 mL of scavenging solution, and 0.05 mL of lipoxygenase solution. In the blank test, the scavenging solution was substituted by 0.15 mL of pure extracting solution. The reaction was spectrophotometrically monitored at 234 nm to follow peroxyl radical conjugate diene formation. The antioxidant activity of enzymatically mediated linoleic acid peroxidation (LIPOX–LINOL) was expressed as the protection percentage monitored by the absorbance at 234 nm, by interpolating the data with those of a calibration curve obtained from standard solutions of Trolox (6-hydroxy-2,5,7,8-tetramethylchromane-2-carboxylic acid). The results were thus reported as mmol of Trolox equivalents (TE)/100 g dried extract.

### 3.5. Statistical Analyses

The general term ‘accession’ commonly used in germplasm collections was adopted throughout this work to equally indicate cultivated forms (cultivars), ecotypes, or natural populations of any evaluated species. Each replication of leaf and flower samples represented the basic experimental unit of each accession.

An analysis of variance (ANOVA) tested the differences between leaves and flowers (across all accessions and replications) for the mean concentration of each of the five groups of phenolics identified in this study and their total, and the mean antioxidant activity determined by three assays (Fremy’s salt scavenging, superoxide anion scavenging and peroxyl radical scavenging). A second ANOVA assessed the differences among the 14 accessions (phenolic concentrations and antioxidant activity assays) for leaves and flowers, individually using the accession × replication variance as the error term. Differences between the areas of origin/sampling of the germplasm (lowlands vs. mountains) were assessed by another ANOVA for leaves and flowers, individually, testing the variance of the ‘area’ factor over the pooled variance of the nested factor ‘accession within area’. In this analysis, the snow clover population #3, originating from the Alps but sampled in the lowland site, was excluded. 

Given the diverse range of *Trifolium* taxa encompassed by the current germplasm, an attempt was made to predict by multiple regression the antioxidant activity in the genus based on the recorded concentrations of phenolic groups. Accession mean values of each of the three antioxidant assays and of the five groups of phenolics were used as dependent variable and independent variables, respectively, in the regressions separately run for leaf and flower data. Prior to the choice of the best predicting models, the presence of multicollinearity between regressors was verified by different indexes, namely, the variance inflation factor, the condition number and the eigenvalues [69]. The choice of the best predicting regression model for each antioxidant assay in leaves and flowers was made examining different methods, namely, the stepwise procedure, the adjusted R^2^, the C(p) index and the Press number, and choosing the most consistent model accordingly [69]. All statistical analyses were carried out using the Proc GLM and Proc Reg of the SAS software.

## Figures and Tables

**Table 1:** List of *Trifolium* germplasm evaluated for the concentration of phenolic compounds and antioxidant activity.

#	Species	Common Name	Germplasm Type	Name/Origin	Adaptation to/Origin From
1	*T. alexandrinum*	Berseem clover	Experimental cultivar	Sintetica E/Italy	Lowlands
2	*T. pratense*	Red clover	Cultivar	Aiace/Italy	Lowlands
3	*T. pratense* subsp. *nivale*	Snow clover	Natural population	Rhaetian Alps	Mountains*
4	*T. repens*	White clover (non-Ladino large-leaved type)	Cultivar	Regal/USA	Lowlands
5	*T. subterraneum*	Subterranean clover	Natural population	Sardinia	Lowlands
6	*T. subterraneum*	Subterranean clover	Natural population	Sardinia	Lowlands
7	*T. pratense*	Red clover	Natural population	Po Valley	Lowlands
8	*T. repens* var. *giganteum*	White clover (Ladino type)	Natural population	Po Valley	Lowlands
9	*T. alpinum*	Alpine clover	Natural population	Graian Alps	Mountains
10	*T. badium*	Brown clover	Natural population	Graian Alps	Mountains
11	*T. ochroleucum*	Sulphur clover	Natural population	Cottian Alps	Mountains
12	*T. pratense* subsp. *nivale*	Snow clover	Natural population	Graian Alps	Mountains
13	*T. repens* var. *sylvestre*	White clover (small-leaved type)	Natural population	Graian Alps	Mountains
14	*T. thalii*	Thal clover	Natural population	Graian Alps	Mountains

* Collected as seed in a mountain area and grown *ex-situ* in a lowland site.

**Table 2:** The most abundant, tentatively identified flavonoids in the 14 *Trifolium* samples and their quantitative evaluation (% dry matter, *n* = 3, mean value ± standard deviation) in leaves and flowers. See Table 1 for taxonomic classification, origin and adaptation of each sample.

#	UV λ_max (nm)_	[M + H]^+^ (*m/z*)	[M − H]^−^ (*m/z*)	MW	Compound	Leaves	Flowers
**1**	253, 354	465, 303	463, 301	464	quercetin-3-*O*-galactoside	-	8.9 ± 0.5
	254, 354	465, 303	463, 301	464	quercetin-3-*O*-glucoside	-	10.2 ± 0.4
	255, 346	449, 287	447, 285	448	luteolin-7-*O*-glucoside	24.8 ± 1.6	8.7 ± 0.2
	266, 432	433, 271	431, 269	432	apigenin-7-*O*-glucoside	4.5 ± 0.3	0.5 ± 0.1
	254, 346	535, 449[M − 86]^+^, 287	533, 489[M − 44]^−^, 285	534	luteolin-7-*O*-glucoside-6”-*O*-malonate	17.8 ± 0.6	30.4 ± 0.9
	266, 337	519, 433[M − 86]^+^, 271	517, 473[M − 44]^−^, 269	518	apigein-7-*O*-glucoside-6”-*O*-malonate	3.8 ± 0.3	2.6 ± 0.4
	254, 344	287	285	286	luteolin	15.3 ± 0.2	3.7 ± 0.9
	260, 324	533, 285	531, 487[M − 44]^−^, 283	532	biochanin A-7-*O*-glucoside-6”-*O*-malonate	7.4 ± 0.7	6.8 ± 0.9
**2**	253, 354	465, 303	463, 301	464	quercetin-3-*O*-galactoside	6.5 ± 0.2	5.5 ± 0.8
	256, 354	551, 303	549, 505[M − 44]^−^, 301	550	quercetin-3-*O*-glucoside-6′′- *O*-malonate	6.2 ± 0.2	14.7 ± 0.5
	265, 344	535, 287	533, 489[M − 44]^−^, 285	534	kaempferol-3-*O*-galactoside-6′′-*O*-malonate	-	11.7 ± 1.3
	251, 300	517, 269	515, 471[M − 44]^−^, 267	516	formononetin-7-*O*-glucoside-6′′-*O*-malonate	14.5 ± 0.8	7.9 ± 0.7
	260, 324	533, 285	531, 487[M − 44]^−^, 283	532	biochanin A-7-*O*-glucoside-6′′-*O*-malonate	20.9 ± 1.6	18.0 ± 1.7
**3**	253, 354	465, 303	463, 301	464	quercetin-3-*O*-galactoside	11.1 ± 0.4	15.1 ± 1.2
	256, 354	551, 303	549, 505[M − 44]^−^, 301	550	quercetin-3-*O*-glucoside-6′′-O-malonate	9.0 ± 0.3	26.3 ± 1.4
	251, 300	431, 269	429, 267	430	formononetin-7-*O*-glucoside	3.1 ± 0.4	1.5 ± 0.2
	251, 300	517, 269	515, 471[M − 44]^−^, 267	516	formononetin-7-*O*-glucoside-6′′-*O*-malonate	3.7 ± 0.1	1.7 ± 0.1
	260, 325	447, 285	445, 283	446	prunetin-4′-*O*-glucoside	7.6 ± 1.5	2.3 ± 0.9
	260, 325	355, 285	531, 487[M − 44]^−^, 283	532	prunetin-4′-*O*-glucoside-6′′-O-malonate	20.0 ± 0.2	9.5 ± 1.2
**4**	266, 337	481, 319	479, 317	480	myricetin-3-*O*-galactoside	-	10.9 ± 0.2
	256, 347	743, 597, 303	741, 301	742	quercetin-3-*O*-{[rhamnosyl-(1→6)]-[xylosyl-(1→2)-galactoside]}	6.4 ± 0.6	2.0 ± 0.1
	256, 354	597, 465, 303	595, 301	596	quercetin-3-*O*-[xylosyl-(1→2)-galactoside]	22.6 ± 2.2	6.8 ± 0.1
	256, 344	727, 581, 449, 287	725, 285	742	kaempferol-3-*O*-{[rhamnosyl-(1→6)]-[xylosyl-(1→2)-galactoside]}	6.3 ± 0.4	1.1 ± 0.1
	253, 354	465, 303	463, 301	464	quercetin-3-*O*-galactoside	3.0 ± 0.2	24.5 ± 0.9
	265, 346	581, 449, 287	579, 285	580	kaempferol-3-*O*-[xylosyl-(1→2)-galactoside]	10.6 ± 0.7	1.5 ± 0.1
	256, 355	507, 303	505, 301	506	quercetin-3-*O*-galactoside-6′′-*O*-acetate	0.9 ± 0.1	23.0 ± 0.3
**5**	259, 325	433, 271	431, 269	432	genistein-7-*O*-glucoside	14.4 ± 0.7	
	265, 344	535, 287	533, 489[M − 44]^−^, 285	534	orobol-7-*O*-glucoside-6′′-*O*-malonate	4.2 ± 0.1	
	259, 325	519, 271	517, 473[M − 44]^−^, 269	518	genistein-7-*O*-glucoside-6′′-*O*-malonate	13.2 ± 0.2	
	260, 325	447, 285	445, 283	446	biochanin A-7-*O*-glucoside	13.5 ± 1.0	
	260, 325	533, 285	531, 487[M − 44]^−^, 283	532	biochanin A-7-*O*-glucoside-6′′-*O*-malonate	35.8 ± 1.9	
**6**	259, 325	433, 271	431, 269	432	genistein-7-*O*-glucoside	11.4 ± 1.3	4.2 ± 0.8
	259, 325	519, 271	517, 473[M − 44]^−^, 269	518	genistein-7-*O*-glucoside-6′′-*O*-malonate	13.6 ± 0.4	6.0 ± 0.4
	251, 300	431, 269	429, 267	430	formononetin-7-*O*-glucoside	4.9 ± 0.7	7.9 ± 1.8
	251, 300	517, 269	515, 471[M − 44]^−^, 267	516	formononetin-7-*O*-glucoside-6′′-*O*-malonate	12.9 ± 0.4	25.5 ± 3.0
	260, 325	447, 285	445, 283	446	biochanin A-7-*O*-glucoside	8.3 ± 0.6	6.7 ± 1.2
	260, 325	533, 285	531, 487[M − 44]^−^, 283	532	biochanin A-7-*O*-glucoside-6′′-*O*-malonate	23.9 ± 2.3	24.8 ± 2.7
**7**	253, 354	465, 303	463, 301	464	quercetin-3-*O*-galactoside	8.7 ± 0.5	8.5 ± 1.2
	256, 354	551, 303	549, 505[M − 44]^−^, 301	550	quercetin-3-*O*-glucoside-6′′-*O*-malonate	8.9 ± 0.2	21.1 ± 0.3
	265, 344	535, 287	533, 489[M − 44]^−^, 285	534	kaempferol-3-*O*-galactoside-6′′-*O*-malonate	-	14.1 ± 2.4
	251, 300	517, 269	515, 471[M − 44]^−^, 267	516	formononetin-7-*O*-glucoside-6′′-*O*-malonate	8.3 ± 0.5	4.7 ± 0.6
	260, 324	533, 285	531, 487[M − 44]^−^, 283	532	biochanin A-7-*O*-glucoside-6′′-*O*-malonate	18.9 ± 1.0	9.4 ± 0.9
**8**	266, 337	481, 319	479, 317	480	myricetin-3-*O*-galactoside	-	13.5 ± 0.6
	256, 347	743, 597, 303	741, 301	742	quercetin-3-*O*-{[rhamnosyl-(1→6)]-[xylosyl-(1→2)-galactoside]}	5.7 ± 0.5	2.1 ± 0.1
	256, 354	597, 465, 303	595, 301	596	quercetin-3-*O*-[xylosyl-(1→2)-galactoside]	22.3 ± 0.6	8.8 ± 0.3
	256, 344	727, 581, 449, 287	725, 285	742	kaempferol-3-*O*-{[rhamnosyl-(1→6)]-[xylosyl-(1→2)-galactoside]}	4.0 ± 0.3	1.3 ± 0.1
	253, 354	465, 303	463, 301	464	quercetin-3-*O*-galactoside	2.5 ± 0.3	29.9 ± 0.5
	256, 355	507, 303	505, 301	506	quercetin-3-*O*-galactoside-6′′-*O*-acetate	1.3 ± 0.1	21.7 ± 0.3
**9**	256, 354	627, 465, 303	625, 301	626	quercetin-hexose-hexose	11.4 ± 0.1	16.6 ± 0.4
	265, 344	611, 449, 287	609, 447, 285	610	kaempferol-hexose-hexose	-	3.6 ± 0.0
	255, 332	949, 625, 463, 303	947	948	302-hexose-hexose-hexose-hexose	10.1 ± 01	1.7 ± 0.1
	256, 355	465, 303	463, 301	464	quercetin-3-*O*-galactoside	0.7 ± 0.0	9.1 ± 0.1
	256, 355	465, 303	463, 301	464	quercetin-3-*O*-glucoside	1.3 ± 0.1	18.9 ± 0.1
	254, 332	833, 303	831, 301	832	302-hexose-hexose-hexose-acetate	6.0 ± 0.2	1.0 ± 0.0
	265, 341	449, 287	447, 285	448	kaempferol-3-*O*-glucoside	-	3.9 ± 0.1
**10**	256, 354	759	757, 595, 462, 301	758	quercetin-hexose-hexose-pentose	5.0 ± 0.1	-
	256, 354	773, 303	771, 609, 462, 301	772	quercetin-hexose-hexose-rhamnose	8.8 ± 0.2	1.0 ± 0.1
	282, 332	463	461, 285	462	unidentified	18.2 ± 0.7	-
	265, 348	449, 287	447, 285	448	luteolin-hexose	6.7 ± 0.4	60.1 ± 0.4
	256, 349	595	593, 447, 285	594	luteolin-rhamnose-hexose	8.7 ± 0.2	8.6 ± 0.1
**11**	256, 354	773, 611, 465, 303	771, 301	772	quercetin-hexose-hexose-rhamnose	3.9 ± 0.1	0.9 ± 0.1
	256, 354	627, 465, 303	625, 301	626	quercetin-hexose-hexose	5.8 ± 0.2	0.3 ± 0.0
	256, 354	611, 449, 303	609, 447, 301	610	quercetin-hexose-rhamnose	6.5 ± 0.2	0.9 ± 0.1
	256, 356	757, 611, 465, 303	755, 301	756	quercetin-hexose-rhamnose-rhamnose	26.7 ± 0.2	10.8 ± 0.5
	256, 355	465, 303	463, 301	464	quercetin-3-*O*-galactoside	8.9 ± 0.2	12.1 ± 0.8
	265, 344	741, 595, 449, 287	739, 593, 447, 285	740	kaempferol-hexose-rhamnose-rhamnose	9.6 ± 0.1	10.5 ± 0.2
	256, 354	551, 303	549, 505[M − 44]^−^, 301	550	quercetin-3-*O*-glucoside-6′′-*O*-malonate	2.4 ± 0.1	26.1 ± 0.1
	265, 344	535, 287	533, 489[M − 44]^−^, 285	534	kaempferol-3-*O*-galactoside-6′′-*O*-malonate	-	16.8 ± 1.9
**12**	253, 354	465, 303	463, 301	464	quercetin-3-*O*-galactoside	4.5 ± 0.3	21.6 ± 1.9
	256, 355	465, 303	463, 301	464	quercetin-3-*O*-glucoside	10.1 ± 2.0	1.1 ± 0.3
	256, 354	551, 303	549, 505[M − 44]^−^, 301	550	quercetin-3-*O*-glucoside-6′′-O-malonate	27.2 ± 2.1	44.6 ± 2.6
	251, 300	431, 269	429, 267	430	formononetin-7-*O*-glucoside	1.8 ± 0.6	-
	260, 325	447, 285	445, 283	446	prunetin-4′-*O*-glucoside	8.7 ± 1.9	0.5 ± 0.0
	260, 325	355, 285	531, 487[M − 44]^−^, 283	532	prunetin-4′-*O*-glucoside-6′′-O-malonate	17.4 ± 1.2	1.9 ± 0.1
**13**	266, 337	481, 319	479, 317	480	myricetin-3-*O*-galactoside	-	18.9 ± 0.2
	256, 354	597, 465, 303	595, 301	596	quercetin-3-*O*-[xylosyl-(1→2)-galactoside]	29.1 ± 0.3	8.1 ± 0.2
	256, 344	727, 581, 449, 287	725, 285	742	kaempferol-3-*O*-{[rhamnosyl-(1→6)]-[xylosyl-(1→2)-galactoside]}	3.3 ± 0.1	0.4 ± 0.1
	253, 354	465, 303	463, 301	464	quercetin-3-*O*-galactoside	3.9 ± 0.1	36.4 ± 0.4
	265, 346	581, 449, 287	579, 285	580	kaempferol-3-*O*-[xylosyl-(1→2)-galactoside]	11.0 ± 1.2	1.1 ± 0.2
	263, 351	449, 287	447, 285	448	kaempferol-3-*O*-galactoside	3.8 ± 0.5	6.3 ± 0.1
	256, 355	507, 303	505, 301	506	quercetin-3-*O*-galactoside-6′′-*O*-acetate	1.5 ± 0.3	17.8 ± 0.3
**14**	266, 337	481, 319	479, 317	480	myricetin-3-*O*-galactoside	-	23.4 ± 0.2
	256, 354	597, 465, 303	595, 301	596	quercetin-3-*O*-[xylosyl-(1→2)-galactoside]	46.2 ± 0.3	10.8 ± 0.3
	253, 354	465, 303	463, 301	464	quercetin-3-*O*-galactoside	4.2 ± 0.2	46.3 ± 0.6
	265, 346	581, 449, 287	579, 285	580	kaempferol-3-*O*-[xylosyl-(1→2)-galactoside]	9.6 ± 0.3	1.2 ± 0.1
	256, 355	523, 319	521, 317	522	myricetin-3-*O*-galactoside-6”-*O*-acetate	-	5.2 ± 0.1
	251, 300	431, 269	429, 267	430	formononetin-7-*O*-glucoside	4.8 ± 0.8	-
	251, 300	517, 269	515, 471[M − 44]^−^, 267	516	formononetin-7-*O*-glucoside-6′′-*O*-malonate	6.0 ± 1.5	-

**Table 3:** Comparison between leaves and flowers (mean values across the 14 *Trifolium* samples reported in Table 1) for concentration of phenolic groups and antioxidant activity.

	Phenolic Group Concentration (mg/g Dry Matter)						Antioxidant Activity Assay		
Sample	Phenolic Acids	Clovamides	Isoflavones	Other Flavonoids	Proanthocyanidins	Total Phenolics	Fremy’s Salt Scavenging *	Superoxide Anion Scavenging *	Peroxyl Radical Scavenging **
leaves	2.13 a	5.23 a	14.51 a	20.83 b	1.12 b	43.83 b	4.94 b	2.03 b	44.93 b
flowers	1.33 a	3.12 a	2.23 b	34.90 a	40.84 a	82.42 a	11.06 a	4.61 a	489.29 a

In each column, mean values followed by different letters (a or b) are different at *P* < 0.05 according to analysis of variance. * μmol gallic acid equivalents (GAE)/100 g freeze-dried matter;.** mmol Trolox equivalents (TE)/100 g freeze-dried matter.

**Table 4:** Concentration of phenolic groups and antioxidant activity determined on leaves of 14 *Trifolium* samples. See Table 1 for taxonomic classification.

	Phenolic Group Concentration (mg/g Dry Matter)						Antioxidant Activity Assay		
#	Phenolic acids	Clovamides	Isoflavones	Other Flavonoids	Proanthocyanidins	Total Phenolics	Fremy’s Salt Scavenging *	Superoxide Anion Scavenging *	Peroxyl Radical Scavenging **
1	4.66	8.53	4.20	41.15	4.10	62.65	5.37	2.12	28.00
2	5.94	29.86	22.63	13.78	0.66	72.89	15.40	4.07	88.25
3	4.68	8.64	16.55	16.88	0.14	46.90	6.08	2.19	21.60
4	1.40	-	0.31	9.19	0.05	10.96	3.18	2.13	10.05
5	2.00	-	70.03	7.55	0.06	79.64	1.47	1.82	137.50
6	1.87	-	48.00	7.94	0.03	57.84	2.15	0.61	8.85
7	2.85	22.33	22.72	21.08	0.12	69.11	9.40	1.92	130.00
8	1.27	-	0.70	6.18	0.25	8.42	3.27	0.60	3.70
9	-	-	0.38	27.68	0.37	28.44	4.49	0.88	16.80
10	-	-	0.17	34.70	8.58	43.45	3.24	5.86	24.30
11	2.42	-	0.54	44.14	0.39	47.51	3.63	2.19	24.10
12	1.71	3.89	14.56	28.54	0.32	49.02	5.54	1.58	110.85
13	-	-	0.56	14.39	0.04	14.95	3.37	0.93	2.55
14	1.02	-	0.79	18.50	0.47	21.79	2.59	1.43	22.45
LSD_(*P* = 0.05)_	1.17	0.48	2.72	2.79	0.18	4.18	2.43	1.60	30.04

* μmol gallic acid equivalents (GAE)/100 g freeze-dried matter; ** mmol Trolox equivalents (TE)/100 g freeze-dried matter.

**Table 5:** Concentration of phenolic groups and antioxidant activity determined on flowers of 13 *Trifolium* samples. See Table 1 for taxonomic classification.

	Phenolic Group Concentration (mg/g Dry Matter)						Antioxidant Activity Assay		
#	Phenolic Acids	Clovamides	Isoflavones	Other Flavonoids	Proanthocyanidins	Total Phenolics	Fremy’s Salt Scavenging *	Superoxide Anion Scavenging *	Peroxyl Radical Scavenging **
1	2.23	6.88	1.15	19.12	29.14	58.53	5.04	4.41	46.30
2	2.63	11.99	10.91	23.70	42.94	92.18	6.20	1.13	423.40
3	2.83	7.29	5.17	22.58	39.03	76.90	5.06	1.00	394.40
4	0.85	-	0.14	15.47	24.10	40.82	9.96	2.69	436.80
6	0.46	-	4.57	0.74	4.96	10.73	2.35	0.76	4.10
7	2.89	10.50	5.44	25.58	37.39	81.81	6.37	1.92	190.00
8	0.44	-	0.08	21.21	30.53	52.27	11.35	1.72	34.60
9	-	-	0.22	27.81	33.31	61.51	8.56	5.48	150.20
10	-	-	-	80.29	81.28	161.57	16.75	9.93	696.00
11	2.56	-	-	44.12	30.30	76.99	7.42	5.31	1065.20
12	1.13	1.29	1.29	54.01	56.88	117.17	9.87	3.67	603.00
13	1.28	-	-	55.60	54.95	111.83	27.19	9.90	940.60
14	-	-	-	63.46	66.11	129.57	27.61	12.01	1376.50
LSD_(*P* = 0.05)_	0.62	0.62	0.62	6.77	3.23	8.93	2.33	2.51	253.37

* μmol gallic acid equivalents (GAE)/100 g freeze-dried matter; ** mmol Trolox equivalents (TE)/100 g freeze-dried matter.

**Table 6:** Mean values of concentration of phenolic groups and antioxidant activity in leaves and flowers of *Trifolium* samples originating from, and sampled in, either lowland. (#1, 2, 4, 5, 6, 7 and 8 in Table 1) or mountain areas (# 9, 10, 11, 12, 13 and 14 in Table 1).

Area of Germplasm Origin and Sampling	Phenolic Group Concentration (mg/g Dry Matter)						Antioxidant Activity Assay		
	Phenolic Acids	Clovamides	Isoflavones	Other Flavonoids	Proanthocyanidins	Total Phenolics	Fremy’s Salt Scavenging *	Superoxide Anion Scavenging *	Peroxyl Radical Scavenging **
*Leaves*									
Lowlands	2.86 a	7.62 a	24.19 a	14.82 b	0.75 b	50.30 a	5.75 a	1.90 a	58.05 a
Mountains	0.86 b	0.65 b	3.00 b	27.99 a	1.70 a	34.19 b	3.81 b	2.15 a	33.51 b
*Flowers*									
Lowlands	1.65 a	5.34 a	4.04 a	11.84 b	28.18 b	57.44 b	6.88 b	2.11 b	189.18 b
Mountains	0.90 b	0.70 b	0.25 b	56.62 a	53.81 a	114.16 a	16.24 a	7.72 a	805.23 a

In each column within each plant organ, mean values followed by different letters (a or b) are different at *P* < 0.05 according to analysis of variance. * μmol gallic acid equivalents (GAE)/100 g freeze-dried matter; ** mmol Trolox equivalents (TE)/100 g freeze-dried matter.

**Table 7:** Prediction of antioxidant activity in leaves and flowers of *Trifolium* germplasm, based on multiple regression analysis of accession mean values of each antioxidant assay on accession mean concentrations of groups of phenolic compounds. Group concentrations in the models (in parentheses) are expressed as mg g^−1^ dry matter.

Antioxidant Activity Assay	Best Linear Model	Model F Probability	Adjusted *R^2^*
*Leaves*			
Fremy’s salt scavenging *	3.368 + 0.373 *(clovamides) − 0.026 *(isoflavones)	< 0.001	0.934
Superoxid anion scavenging *	1.194 + 0.059 *(clovamides) + 0.468 *(proanthocyanidins)	< 0.001	0.685
Peroxyl radical scavenging * *	−2.217 − 12.624 *(phenolic acids) + 4.012 *(clovamides) + 1.317 *(other flavonoids) + 1.765 *(isoflavones)	< 0.05	0.554
*Flowers*			
Fremy’s salt scavenging *	5.444 − 1.886 *(phenolic acids) + 0.233 *(other flavonoids)	< 0.001	0.524
Superoxid anion scavenging *	0.887 − 0.230 *(clovamides) + 0.127 *(other flavonoids)	< 0.001	0.728
Peroxyl radical scavenging **	−242.1 + 217.8 *(phenolic acids) − 80.8 *(clovamides) + 16.5 *(other flavonoids) + 52.5 *(isoflavones)	< 0.05	0.601

* μmol gallic acid equivalents (GAE)/100 g freeze-dried matter; ** mmol Trolox equivalents (TE)/100 g freeze-dried matter
